# Cannabinoid-Mediated Inhibition of Recurrent Excitatory Circuitry in the Dentate Gyrus in a Mouse Model of Temporal Lobe Epilepsy

**DOI:** 10.1371/journal.pone.0010683

**Published:** 2010-05-17

**Authors:** Muthu D. Bhaskaran, Bret N. Smith

**Affiliations:** 1 Department of Physiology, University of Kentucky, Lexington, Kentucky, United States of America; 2 Department of Cell and Molecular Biology, Tulane University, New Orleans, Louisiana, United States of America; Vrije Universiteit Amsterdam, Netherlands

## Abstract

Temporal lobe epilepsy (TLE) is a neurological condition associated with neuron loss, axon sprouting, and hippocampal sclerosis, which results in modified synaptic circuitry. Cannabinoids appear to be anti-convulsive in patients and animal models of TLE, but the mechanisms of this effect are not known. A pilocarpine-induced status epilepticus mouse model of TLE was used to study the effect of cannabinoid agonists on recurrent excitatory circuits of the dentate gyrus using electrophysiological recordings in hippocampal slices isolated from control mice and mice with TLE. Cannabinoid agonists WIN 55,212-2, anandamide (AEA), or 2-arachydonoylglycerol (2-AG) reduced the frequency of spontaneous and tetrodotoxin-resistant excitatory postsynaptic currents (EPSCs) in mice with TLE, but not in controls. WIN 55,212-2 also reduced the frequency of EPSCs evoked by glutamate-photolysis activation of other granule cells in epileptic mice. Secondary population discharges evoked after antidromic electrical stimulation of mossy fibers in the hilus were also attenuated by cannabinoid agonists. Agonist effects were blocked by the cannabinoid type 1 receptor (CB1R) antagonist AM251. No change in glutamate release was observed in slices from mice that did not undergo status epilepticus. Western blot analysis suggested an up-regulation of CB1R in the dentate gyrus of animals with TLE. These findings indicate that activation of CB1R present on nerve terminals can suppress recurrent excitation in the dentate gyrus of mice with TLE. This suggests a mechanism for the anti-convulsive role of cannabinoids aimed at modulating receptors on synaptic terminals expressed de novo after epileptogenesis.

## Introduction

Development of temporal lobe epilepsy (TLE) is often associated with gliosis, neuron loss, axon sprouting, and synaptic reorganization in temporal lobe brain structures. These changes occur variably in concert with the development of spontaneous recurrent seizures in humans and in animal models of inducible TLE. In particular, mossy fiber sprouting in the dentate gyrus is a widely studied correlate of adult TLE development [1], [2], [3], [4], [5], [6], [7]. A dominant hypothesis regarding the outcome of this structural metamorphosis is that newly sprouted mossy fibers form recurrent synaptic connections with other granule cells, thus accounting for increased excitability of the dentate gyrus under experimental conditions [8], [9], [10], [11], [12], [13]. The dentate gyrus is particularly amenable to study in TLE models because of the well-defined nature of the anatomical and functional changes, but synaptic reorganization and recurrent excitation likely also occur in other regions [7], [14], [15], [16], [17]. The well-described changes in the dentate gyrus may serve as a model for altered synaptic activity in other affected brain areas.

Cannabinoids have been implicated for the treatment of several neurological disorders associated with neural excitability changes [18], [19], [20], [21]. However, using cannabinoids to treat epilepsy has been controversial [22]. In the brain, cannabinoids act mainly at G protein-coupled, cannabinoid type 1 receptors (CB1R) [23]. The usual cellular location for the CB1R in the hippocampus and other brain regions is synaptic terminals of GABAergic interneurons and select glutamatergic principal cells [24], [25], [26], [27], [28]. Typically, binding CB1R diminishes synaptic inhibition or excitation by suppressing GABA or glutamate release, respectively. The presence of CB1R on glutamatergic terminals might thus contribute to anti-epileptic activity of cannabinoids by reducing glutamate release, whereas receptors located on GABA terminals might exacerbate seizures by limiting GABA release. Regional CB1R expression changes have been detected in tissue from human patients and animal TLE models [29], [30]. Systemic cannabinoid treatment suppresses seizures and increases seizure threshold in epileptic rats [31], whereas CB1R antagonists cause seizure-like activity in hippocampal culture models of acquired epilepsy [32] and treatment with CB1R antagonists may exacerbate seizures in patients with TLE [33]. Cannabinoid-mediated responses that are consistent with their putative anti-convulsive actions have not been adequately investigated, and cellular effects on synaptically-reorganized tissue from epileptic animals are unknown. We therefore tested the hypothesis that glutamate release is suppressed by CB1R activation in the reorganized dentate gyrus granule cell layer of animals with TLE.

## Methods

### Ethics Statement

All procedures were carried out in accordance with NIH Guidelines and were approved by the Tulane University and University of Kentucky Animal Care and Use Committees.

### Animals and Pilocarpine-injection

Adult male CD1 mice weighing 25–30g were housed individually on a 12-hour day/night cycle. Food and water were available ad libitum. The animals were housed at least 7 days prior to treatment. Mice were administered an intraperitoneal (i.p.) injection of methylscopolamine (1 mg/kg) in sterile saline vehicle (0.9% NaCl; 0.1 ml total volume) 15–30 min prior to injection of pilocarpine to reduce the peripheral cholinergic effects of the pilocarpine. Experimental animals were then injected i.p. with a single dose of pilocarpine (280–290 mg/kg) as described previously [7]. Control mice were age-matched with treated mice and were administered a comparable volume of vehicle or were not injected after the initial methylscopolamine treatment.

Seizure behavior was observed for at least 2 hours starting immediately after the pilocarpine injection. The category and the number of generalized convulsive seizures in each ½ hour period were tallied. A modified version of the seizure scale described by Racine [34] was used to identify seizure severity. Special attention was given to convulsive seizures (i.e., categories 3 to 5) as they were correlated previously with the eventual development of spontaneous seizures and mossy fiber sprouting [7], [8]. Convulsive seizures most often included unilateral limb myoclonus, loss of postural control and repetitive jumping. Seizures were typically of 30–90s duration and were separated by periods of relative inactivity of variable duration. The periods between convulsive seizures were marked by continuous low-level seizure-like activity (i.e., categories 1-2) that typically included continuous head bobbing, chewing, stiff tail, and intermittent wet dog shakes. A mouse that experienced a minimum of 3 generalized convulsive seizure events within 2 hours following pilocarpine injection was considered to have undergone status epilepticus (SE). In addition to standard rodent chow and water, mice were supplied with water-moistened chow and a 5% sucrose solution in water in a petri dish inside the cage for at least 4 days after SE induction to help replenish fluids. Mice were housed individually for 4–10 weeks after pilocarpine-induced SE prior to electrophysiological analysis. Seizure activity was assessed by passive observation for 4–6 hr/wk beginning 3 weeks after SE. Once spontaneous seizures were detected, animals that survived SE became candidates for in vitro experiments. In the event no seizures were observed, animals were used at 10 weeks post-treatment.

### Slice preparation and electrophysiological recording

Mice were anesthetized by halothane inhalation and then decapitated. The brains were rapidly removed and immersed in oxygenated (95% O_2_/5% CO_2_) ice-cold (0–4°C) artificial cerebrospinal fluid (ACSF) containing (in mM): 124 NaCl, 3 KCl, 26 NaHCO_3_, 11 glucose, 2 CaCl_2_, and 1.3 MgCl_2_, pH = 7.3–7.4, with an osmolality of 290–305 mOsm/kg. Brains were then blocked and glued to a sectioning stage and transverse slices (300 µm) of the hippocampal formation (i.e., approximately horizontally through the ventral 2/3 of the hippocampal formation) were cut in cold oxygenated ACSF using a vibrating microtome (Vibratome Series 1000; Technical Products Intl, St Louis, MO, USA). The hippocampus was then separated from the surrounding tissue, being sure to *completely* remove the entorhinal cortex. Slices were then transferred to a storage chamber, where they were immersed in warm (32–35°C) and oxygenated ACSF.

Extracellular field potential recordings: Field-potential recordings were obtained from granule cells in the dentate gyrus using a glass micropipette containing 1M NaCl placed near the apex of the granule cell layer. Voltage signals were recorded using an Axopatch 1D amplifier (Axon Instruments; Union City, CA), low-pass filtered at 2–5 kHz, digitized at 88 kHz (Neurocorder, Cygnus Technology, Delaware Water Gap, PA), stored on videotape, and acquired to a PC-style computer using a Digidata 1320A and pClamp programs (Clampex 8; Axon Instruments, Union City, CA, USA).

Whole-cell patch-clamp recordings: After an equilibration period of 1–2h, granule cells in the dentate gyrus were targeted for recording under a 40× water-immersion objective (NA  =  0.8) with infrared-differential interference contrast (IR-DIC) optics (Olympus BX-51WI) using a CCD camera. Whole-cell voltage-clamp recordings from dentate granule cells were made using patch pipettes with open resistances of 2–5 MΩ using a Multiclamp 700A (Molecular Devices, Sunnyvale, CA, USA) or Axopatch 200B amplifier (Axon Instruments). Signals were low-pass filtered at 2–5kHz, digitized at 88kHz (Neurocorder, Cygnus Technology, Delaware Water Gap, PA, USA), and recorded onto videotape as well as to a PC-style computer (Digidata 1320A or 1440A; Axon Instruments or Molecular Devices). Data were captured using pCLAMP programs (Axon Instruments or Molecular Devices) and analyzed using pCLAMP or Mini-analysis (Synaptosoft, Decatur, GA, USA).

Patch pipettes were pulled from borosilicate glass (Garner Glass Co., Claremont, CA, USA) and were filled with an intracellular solution containing (in mM): 130 K^+^-gluconate, 10 HEPES, 1 NaCl, 1 MgCl_2_, 1 CaCl_2_, 3 KOH, 5 EGTA, and 2–4 Mg-ATP. Seal resistance was typically 1–4 GΩ and series resistance, measured from brief voltage steps (10 mV, 5 ms) applied through the recording pipette was typically <20 MΩ, uncompensated. Recordings in which a >20% change in series resistance was measured during the recording were excluded from the analysis. Input conductance was estimated by measuring the current at the end of brief (20–400 ms) voltage pulses of 5–10 mV. Resting membrane potential (−8 mV correction for liquid junction potential) was determined by periodically monitoring the voltage at which no current was measured (i.e. briefly removing voltage-clamp control of the neuron by switching to *I* = 0) during the recording.

Electrical stimulation of mossy fibers was made using a concentric bipolar platinum-iridium electrode (125 µm diameter; FHC, Inc. Bowdoinham, ME) placed in the hilus. Stimulation intensity was initially adjusted to the minimum intensity required to evoke a single antidromic population spike, which was considered to be the threshold stimulation intensity (T). Stimulation-response characteristics were obtained for each slice using increasing multiples of T. At least 5 stimuli at 20–120s intervals were made at each multiple of T.

Chemical activation of intact granule cell circuits was done by photolytic “uncaging” of glutamate in the dentate gyrus (i.e., glutamate photoactivation), similar to previous descriptions [8], [12], [35]. L-glutamatic acid, γ-(-carboxy-2-nitrobenzyl) ester, trifluoroacetic acid salt (i.e., CNB-caged glutamate, 250 µM; Invitrogen, Carlsbad, CA, USA), which does not bind glutamate receptors, was added to re-circulating ACSF and was “uncaged” using brief exposure to UV light. Epifluorescence illumination through a UV filter (excitation, 360–70 nm; emission long pass, 420 nm; dichroic, 400 nm; Chroma Technology, Rockingham, VT) was used to direct UV light into the slice through the 40x objective used to obtain the recording, which was moved progressively further away from the recorded cell until a photolysis-mediated increase in synaptic events was found in the absence of a direct inward glutamate-mediated current. Exposure time (10 ms) was electronically controlled using a Uniblitz shutter (Vincent Associates, Rochester, NY) and apertures along the light path confined the diameter of effective uncaging to approximately 50 µm. Opening the shutter with no UV filter or with other filters in place (e.g., FITC) did not result in uncaging. Glutamate was uncaged in at least five points along the length of the granule cell layer, including the apex and the tips of the supra- and infrapyramidal blades, as well as in the hilus and proximal CA3 pyramidal regions.

### Drug application

Population and whole cell recordings were usually done in the absence of Mg^2+^ in the ACSF in order to remove the Mg^2+^-dependent blockade of the NMDA receptors and expose circuit excitability [8], [15]. Added to the ACSF for specific experiments were the GABA_A_ receptor antagonist, bicuculline methiodide (30 µM; Sigma), tetrodotoxin (TTX; 1–2 µM; Sigma or Alomone labs, Jerusalem, Israel), the glutamate AMPA/kainate receptor antagonist 6-cyano-7-nitroquinoxaline-2,3-dione (CNQX; 10 µM; Sigma), and the NMDA receptor antagonist DL-5-aminophosphonovaleric acid (AP-5; 50 µM; Sigma). The synthetic cannabinoid agonist (*R*)-(+)-[2,3-Dihydro-5-methyl-3[(4-morpholinyl)methyl]pyrrolo[1,2,3-de]-1,4-benzoxazinyl]-(1-naphthalenyl)methanone (WIN55,212-2; 1–10 µM; Sigma), the endogenous cannabinoid ligands, arachidonoylethanolamide (anandamide, AEA; 1–10 µM; Tocris) and 2-arachidonylglycerol (2-AG; 1-10 µM; Tocris), and the CB1R antagonist/inverse agonist, N-(Piperidin-1-yl)-5-(4-iodophenyl)-1-(2,4-dichlorophenyl)-4-methyl-1*H*-pyrazole-3-carboxamide (AM-251; 10 µM; Tocris) were used to activate and block CB1R-mediated responses, respectively. In general, antagonists and toxins were added continuously for at least 20 min prior to response measurements; agonists were bath applied for 5–10 min.

### Tissue staining

At the end of the experiment, slices were placed in 0.37% sodium sulfide solution in 0.1M NaHPO_4_ buffer for 25 minutes and then fixed overnight in 4% paraformaldehyde. The slices were then rinsed with phosphate buffered saline (PBS; 0.01M) and placed in 30% sucrose for cryoprotection. After equilibration with sucrose, 30 µm frozen sections were made using a cryostat, mounted on charged slides (Superfrost Plus; Fisher Scientific), and air-dried overnight. Timm's and Nissl staining was performed to reveal mossy fiber and cellular distribution patterns using the protocols used previously [7]. Timm-stained sections were assessed semi-quantitatively using the rating scale of Tauck and Nadler [4], where a score of zero indicated little or no Timm staining in the granule cell layer; 1 indicated mild, patchy staining in the granule cell layer; 2 indicated moderate continuous staining through the granule cell layer with discontinuous, punctate staining in the inner molecular layer; and 3 indicated a continuous band of intense staining in the inner molecular layer. Mossy fiber sprouting scores are reported as the average of assessments from five to eight sections per animal.

### Western Blot detection and quantification

Transverse sections of the hippocampus (400 µM thickness) were made from control and pilocarpine-treated mice that survived status epilepticus using a vibratome, and the dentate gyrus was micro-dissected to include the molecular layer. The samples were homogenized at 4°C in lysis buffer (0.5M HEPES, 3M NaCl, 1M MgCl_2_, 0.5M EDTA, 0.1M DTT, 10% SDS, 10% deoxycholate and 0.3% Triton X-100) and centrifuged at 13000 rpm at 4°C for 10 minutes. The supernatant was collected leaving the pellet behind. Proteins were quantified using Bradford protein assay. Each lane was loaded with 15 µg of protein in 10% Tris·HCl polyacrylamide gels and electrophoresed at 110 V for 60 to 90 min. Proteins were then transferred at 200 mA for 1h to polyvinylidene difluoride membranes for Western blot analysis. Membranes were rinsed in PBS and blocked in 1∶1 Odyssey blocking buffer/PBS (Odyssey, Li-COR Biosciences, Lincoln, NE) for 1h at room temperature. Membranes were then either incubated 1hr at room temperature or overnight at 4°C, with a rabbit anti-rat CB1R (1∶2500) (Alpha Diagnostic International, San Antonio, TX) and a rabbit anti-mouse α-actin (1∶1000) primary antiboidies (Sigma, St.Louis, MO) in 1∶1 Odyssey blocking buffer/PBS/0.1% Tween 20. Following four 5-min washes (PBS + 0.1% Tween 20); membranes were incubated with fluorescence-conjugated goat anti-rabbit IRDye-680® and goat anti-rabbit IRDye-800® secondary antibodies (1∶5000 Odyssey) in Odyssey blocking buffer/PBS/0.1% Tween 20 and 0.01% SDS for 1 hr, followed by four 5-min washes. After a final 10 min PBS wash, the membranes were dried and blots were scanned by a densitometer (Odyssey model 9120, Li-COR Biosciences, Lincoln, NE) to quantify band density. Background density was subtracted from the CB1R band density and normalized to α-actin, which was used as the loading control.

### Statistical analysis

A value of twice the mean peak-to-peak noise level for a given recording in control solutions was used as the detection limit for minimal PSC amplitude (i.e., typically 5–10 pA). For sEPSCs and mEPSCs, at least 2 min of activity in control and in agonist-containing ACSF was examined to identify cannabinoid effects on amplitude and frequency. The effects of drugs on sEPSC and mEPSC frequencies were analyzed within a recording using the Kolmogorov-Smirnov (K-S) test. Typically, 100–300 events were compared for each condition. Pooled results from responding cells were analyzed using a paired Student's *t*-test. For uncaging experiments, the data collected within a given recording were analyzed using Kruskal-Wallis test. Western blots were analyzed using single factor ANOVA. Results are reported as the mean ± SEM unless indicated otherwise; significance was set at significance was set at *P*<0.01 for the K-S test and *P*<0.05 for all other statistical measures.

## Results

### Histology and synaptic activity in slices from epileptic mice

**Figure 1:**
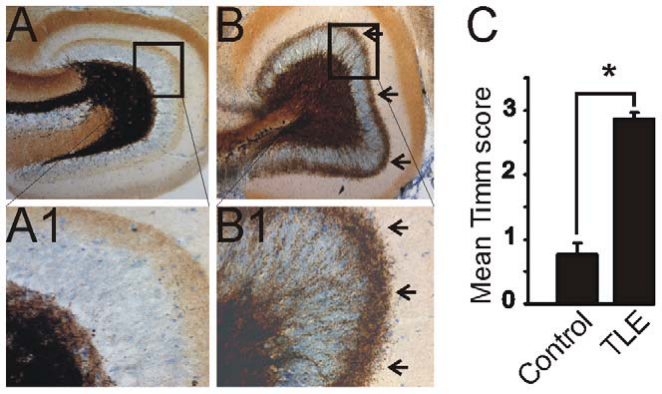
Timm stain illustrating mossy fiber sprouting into the dentate gyrus inner molecular layer in pilocarpine-treated mice. **A.** Dentate gyrus of a normal mouse. **B.** Dentate gyrus of a pilocarpine-treated mouse that survived status epilepticus (SE) showing mossy fiber sprouting. **A1** and **B1** are enlarged boxed regions of **A** and **B**. The arrows in **B** and **B1** point to extensive mossy fiber sprouting into the inner molecular layer of the dentate gyrus. **C.** Graph showing the mean Timm score between controls and pilocarpine-treated mice that survived SE. Asterisk, significant difference between mean scores (P<0.05).

As in previous analyses of this model, animals that underwent SE after pilocarpine injection developed spontaneous seizures, had mossy fiber sprouting, and showed evidence of recurrent excitatory circuit formation in the dentate gyrus, whereas animals that did not undergo SE were normal [7], [8]. Similar to those previous analyses, no major differences in mossy fiber sprouting or synaptic responsiveness were observed between animals examined at different times post-treatment within the six seek time-frame for these studies. Figure 1 illustrates Timm's staining in an animal that underwent SE and developed spontaneous seizures compared to a control animal. Mean Timm scores were 0.76±0.19 for 50 sections from 10 control mice and 2.87±0.08 for 45 sections from 9 pilocarpine-treated mice that survived SE.

Extracellular field and whole-cell patch-clamp recordings were made from 21 untreated animals, 11 pilocarpine-treated animals that did not undergo SE (injected controls) and 39 pilocarpine-treated animals that survived SE and experienced at least one spontaneous seizure. Preliminary tests were done to detect differences between cells from injected controls and untreated animals. Since the untreated animals and the injected controls did not differ behaviorally or physiologically, data from these animals were combined and considered as control data unless otherwise noted.

**Figure 2:**
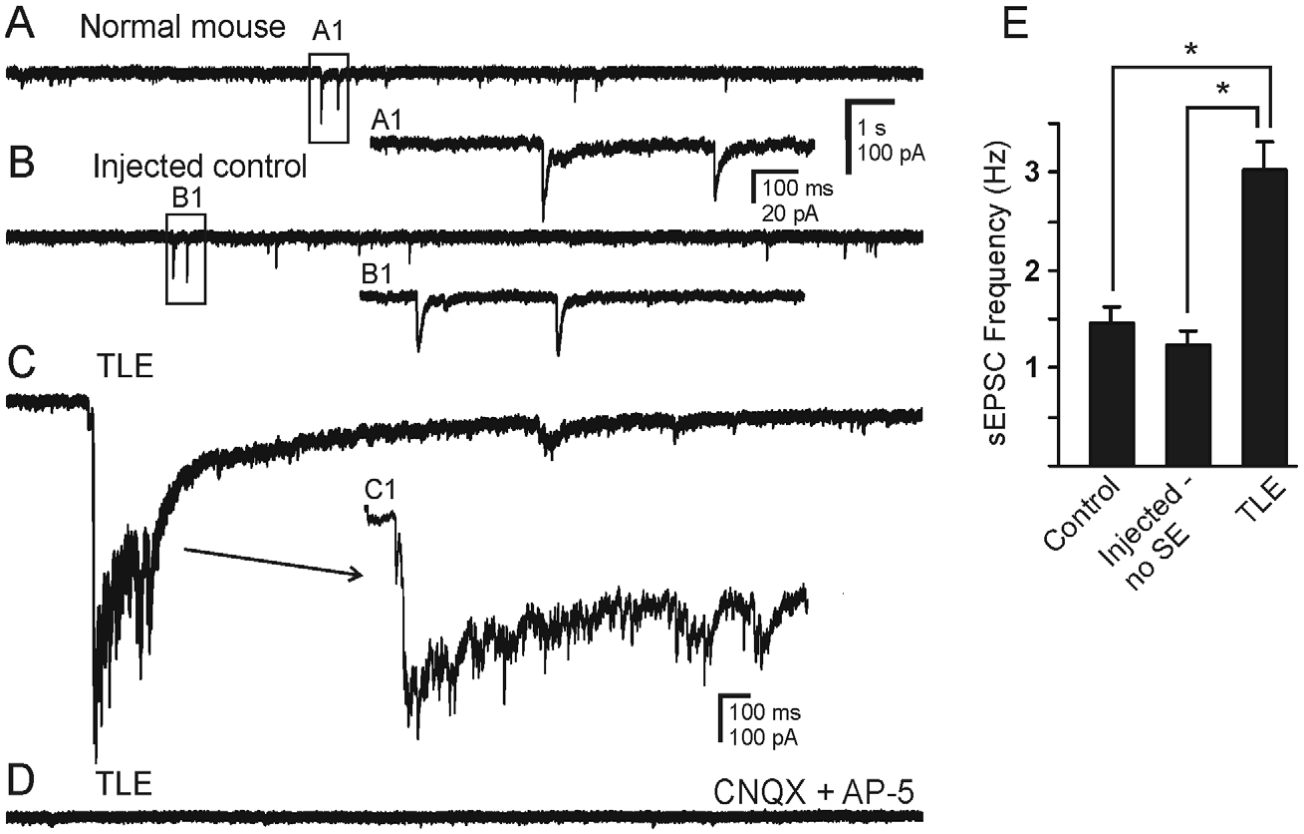
Increase in synaptic activity in granule cells from pilocarpine-treated mice that survived SE and developed TLE. **A**. sEPSCs recorded from granule cell of normal mouse. **B**. sEPSCs recorded from granule cell of a mouse that was injected with pilocarpine but did not develop SE. **C**. sEPSCs and bursting activity recorded from a granule cell of a pilocarpine-treated mouse that developed TLE. **D**. Trace showing that sEPSCs were blocked by application of ionotropic glutamate receptor antagonists, CNQX (10 µM) and AP-5 (50 µM). **A1** and **B1** are expanded boxed regions of **A** and **B**. **C1**, expansion of the sEPSC showing increased synaptic activity during the burst seen in **C**. Recordings were made in the absence of added Mg^2+^ and the presence of bicuculline (30 µM). **E**. Bar graph comparing the frequency of sEPSCs among the three groups of animals. Asterisk indicates significantly higher frequency in cells from mice with TLE (P<0.05).

In nominally Mg^2+^-free ACSF that contained bicuculline (30 µM), a solution that was meant to expose multisynaptic glutamatergic connectivity and reduce recurrent GABAergic inhibition, patch-clamp recordings from granule cells in normal animals (n = 10) and in injected controls (n = 9) revealed sEPSCs with an average frequency of 1.45±0.17 Hz and 1.24±0.15 Hz, respectively (Fig. 2; P>0.05). In animals that had experienced SE and developed spontaneous seizures, barrages of sEPSCs were superimposed on the ongoing sEPSC activity (Fig. 2). The frequency of sEPSCs in cells from these animals, including those within the sEPSC barrages, was 3.03±0.28 Hz (n = 9), which was significantly higher than in either of the control groups (P<0.05). Within a given recording, these values varied little over time, but variability between cells could be large. Application of glutamate antagonists CNQX (10 µM) and AP5 (50 µM) blocked the sEPSCs and sEPSC barrages (n = 3; Fig 2).

**Figure 3:**
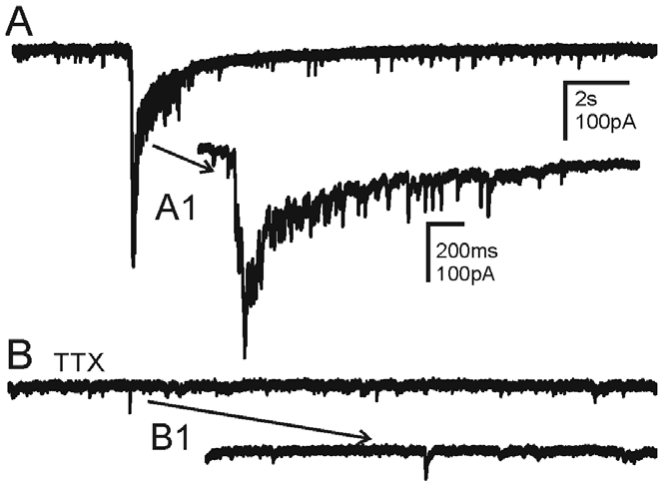
Action potential dependence of epileptiform EPSC bursts. **A**. Spontaneous burst activity recorded from a granule cell in a pilocarpine-treated mouse that survived SE. **B**. The bursts were completely blocked by TTX (1 µM), indicating they were action potential-dependent. Action potential-independent mEPSCs were still seen in the presence of TTX. **A1** and **B1** are expanded segments of **A** and **B** respectively. The recordings were made in the absence of Mg^2+^ and presence of bicuculline (30 µM).

Barrages of sEPSCs were also prevented by addition of TTX (1–2 µM; Fig. 3), which blocked action potential dependent synaptic release but did not prevent action potential-independent miniature EPSCs (mEPSCs), suggesting the barrages were due to action potentials in afferent neurons. We also determined whether action potential-independent glutamate release was different between the two groups. In the presence of TTX, mEPSC frequency in controls was 1.14±0.13 Hz (n = 14) and in pilocarpine-treated mice was 2.41±0.26 Hz (n = 18; P<0.05; unpaired t-test). These data suggested that granule cells from pilocarpine-treated mice that survived SE received enhanced spontaneous glutamatergic synaptic input versus control mice or mice that did not experience SE after pilocarpine injection. Activity of local glutamatergic neurons and of additional synaptic contacts contributed to the enhanced glutamate release.

### Effect of CB1R activation on sEPSCs

**Figure 4:**
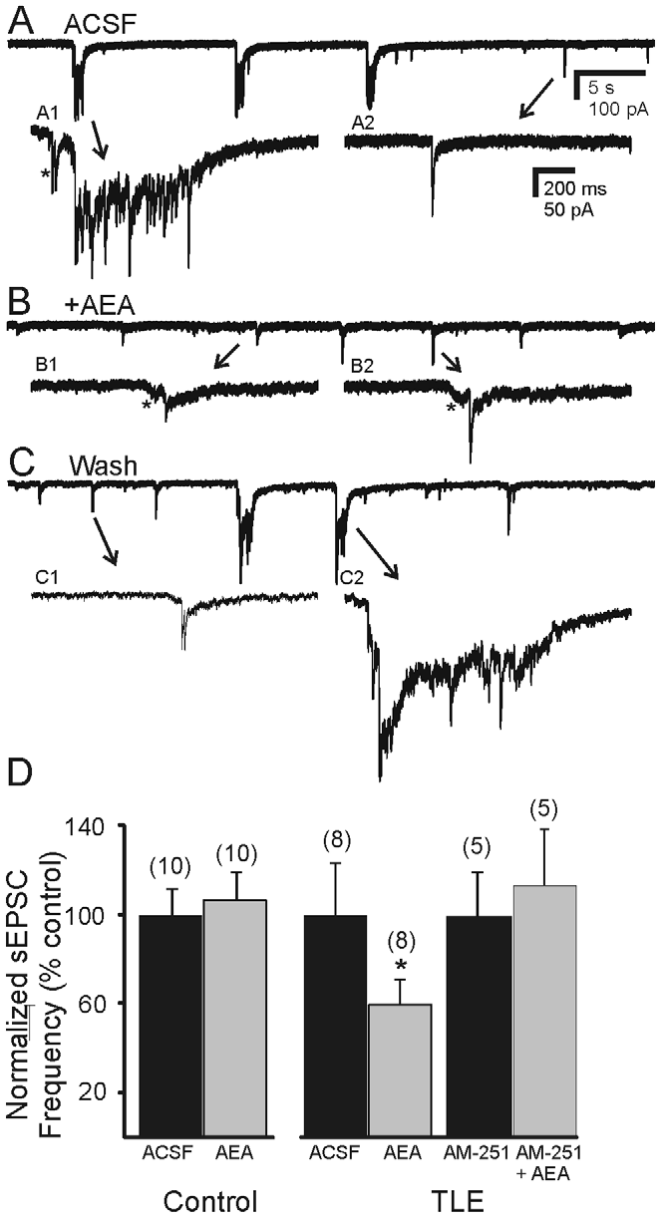
Inhibitory effect of anandamide (AEA) on sEPSCs. **A**. Spontaneous bursts of EPSCs observed in a granule cell from a pilocarpine-treated mouse that survived SE. **A1** and **A2** are expanded segments of the events indicated by the arrows. **B**. AEA (10 µM) suppressed the epileptiform bursts of activity. **B1** and **B2** are examples of individual events from **B**. **C**. Bursts were reinstated after 20 minute wash to normal ACSF. **C1** and **C2**, expanded segments of **C**. Asterisks indicate currents preceding the largest amplitude EPSCs in some cases. The recordings were made in the absence of Mg^2+^ and the presence of bicuculline (30 µM). **D**. Cumulative graph of normalized sEPSC frequency from control mice and pilocarpine-treated mice that survived SE and developed TLE before and after application of AEA. Asterisk in **D** indicates significant reduction in frequency by AEA (P<0.05). In mice with TLE, the effect of AEA was prevented by preapplication of the CB1 receptor antagonist AM-251 (10 µM). Number of cells for each experiment is in parentheses.

In Mg^2+^-free ACSF containing bicuculline, application of AEA (1–10 µM) to recordings from granule cells in control animals resulted in no change in amplitude or frequency of sEPSCs (1.46±0.17 Hz control; 1.55±0.18 Hz AEA; P>0.05; n = 10). In granule cells from mice that survived pilocarpine-induced SE, application of AEA reduced the frequency of sEPSCs from 3.02±0.7 Hz in control ACSF to 1.80±0.35 Hz in AEA (Fig. 4; P<0.05; n = 8). In addition, the occurrence of sEPSC bursts was eliminated by AEA. Application of AEA thus suppressed EPSC bursting and frequency in these animals. The inhibitory effect of AEA was prevented by pre-application of the CB1R antagonist AM-251 (10 µM) (2.60±0.52 Hz to 3.06±0.67; P>0.05; n = 5; Fig 4D), indicating the involvement of CB1R in the response. No significant effect of AM-251 alone on sEPSC frequency was observed. Application of the synthetic cannabinoid agonist WIN 55,212-2 (1 µM) had similar effects on sEPSC bursts and reduced the frequency of sEPSCs from 2.77±0.67 Hz to 1.73±0.43 Hz (5 of 5 cells; P<0.05). Cannabinoid agonists acting at CB1R suppressed sEPSC bursts in pilocarpine-treated mice that survived SE, but had no effect on overall excitatory synaptic input in control mice.

### Effect of CB1R activation on mEPSCs

**Figure 5:**
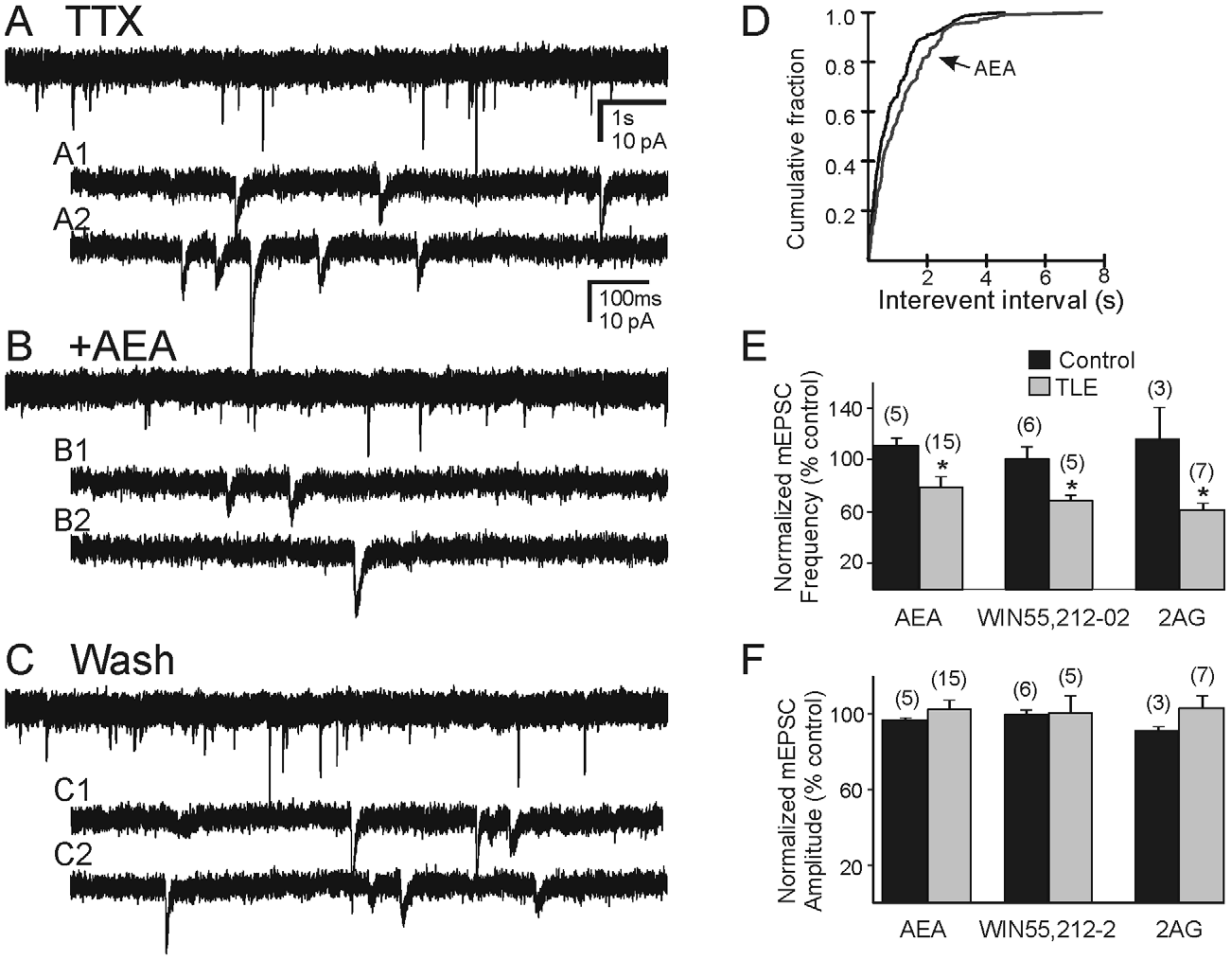
Effect of cannabinoid agonists on TTX-independent mEPSCs in pilocarpine-treated mice that survived SE. **A**. mEPSCs recorded from a granule cell in the presence of TTX (1 µM). **B**. Trace showing the reduction of mEPSC frequency after application of AEA (10 µM). **C**. Wash to TTX alone (20 min) showing that the effect was reversible. **A1**, **A2**, **B1**, **B2** and **C1**, **C2** are expanded segments of **A**, **B** and **C**, respectively. Recordings were made in the presence of bicuculline (30 µM). **D**. Cumulative probability plot showing a change in the frequency of mEPSCs in this cell. **E and F**. Cumulative charts showing effect of AEA, WIN 55,212-2 and 2-AG on mEPSC frequency (**E**) and amplitude (**F**) on pilocarpine-treated (TLE) and control mice. Asterisk indicates significant change in mEPSC frequency (p<0.05); number of recordings shown in parentheses.

To determine if the effect of CB agonists was due to binding of receptors on presynaptic terminals, we determined the effect of AEA on mEPSCs in the presence of TTX. In control animals, AEA did not change the frequency of mEPSCs (1.35±0.25 Hz control vs 1.25±0.17 Hz in AEA; n = 5; p>0.05). Application of AEA (10 µM) in pilocarpine treated mice that survived SE significantly reduced the frequency of mEPSCs from 2.53±0.40 Hz prior to drug application to 1.81±0.22 Hz after drug application (n = 15; P<0.05; Fig. 5). Within 10 min of application, the frequency of mEPSCs was decreased by AEA in 8 neurons and unchanged in three cells. In the remaining four cells, the frequency was transiently increased within the first 3 min of AEA application. The amplitude of mEPSCs was not affected by AEA in either group (P>0.05; Fig. 5F).

Since AEA can have effects at non-CB receptors (i.e., TRPV1), effects of relatively selective CB agonists were assessed. Similar effects to those of AEA were observed when the synthetic CB agonist WIN 55,212-2 (1-10 µM) or the endogenous cannabinoid 2-AG (10 µM) were applied (Fig. 5E,F), except that transient increases in mEPSC frequency were not observed. The mEPSC frequency in pilocarpine-treated mice was reduced from 2.13±0.23 Hz to 1.45±0.15 Hz (n = 5; P<0.05) by WIN 55,212-2 (10 µM), while 2-AG reduced the frequency from 2.18±0.52 Hz to 1.27±0.26 Hz (n = 7; P<0.05). Neither drug changed the mEPSC amplitude significantly (P>0.05). The frequency of mEPSCs in granule cells was thus suppressed by cannabinoid agonists in mice that had survived pilocarpine-induced SE, but not in control mice or in mice that did not undergo SE.

### Effect of CB1R activation on population responses

**Figure 6:**
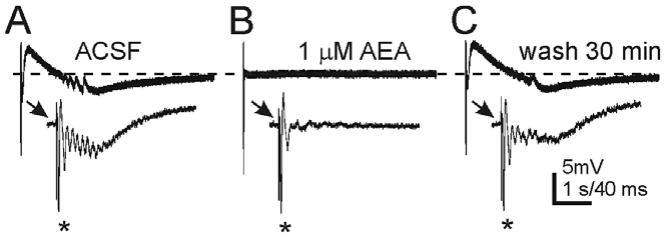
Anandamide attenuated secondary population activity that followed antidromic stimulation of granule cell axons. **A**. Trace showing granule cell response to mossy fiber stimulation in a mouse with TLE. **B**. Antidromically-evoked secondary afterdischarges were reduced in the presence of 1 µM AEA. **C**. The effect of AEA was reversible after 30 min wash to control ACSF. Recordings were made in the absence of Mg^2+^ and the presence of 30 µM bicuculline; stimulus intensity 0.1 mA.

To determine if coordinated granule cell population activity was also suppressed by CB1R activation, antidromically-evoked population responses to electrical stimulation of mossy fibers in the hilus, and the effects of cannabinoids on those responses were assessed. Population activity in slices from control animals was composed of a single antidromic population spike, whose amplitude was increased with increasing stimulus intensity, as described previously [8]. Application of AEA (1–10 µM; n = 5) or WIN 55,212-2 (10 µM; n = 3) had no effect on the responses in slices from control mice. In slices from mice with TLE, responses were more robust, usually consisting of an antidromic population spike followed by a secondary after-discharge, often of long and variable latency after the initial antidromic response [8], [9]. In slices from these animals, application of AEA (n = 10; Fig. 6) or WIN 55,212-02 (n = 5) suppressed the secondary after-discharge, with no effect on the antidromic population spike amplitude. This effect was prevented in each of 6 slices by pre-application of AM-251 (10 µM), indicating it was mediated by CB1R activation. In addition to suppressing synaptic activity, CB1R activation suppressed the secondary population afterdischarge in the dentate gyrus of mice with pilocarpine-induced TLE, suggesting effects on recurrent excitatory circuits.

### Activation of recurrent excitatory circuit by photolysis of caged glutamate

**Figure 7:**
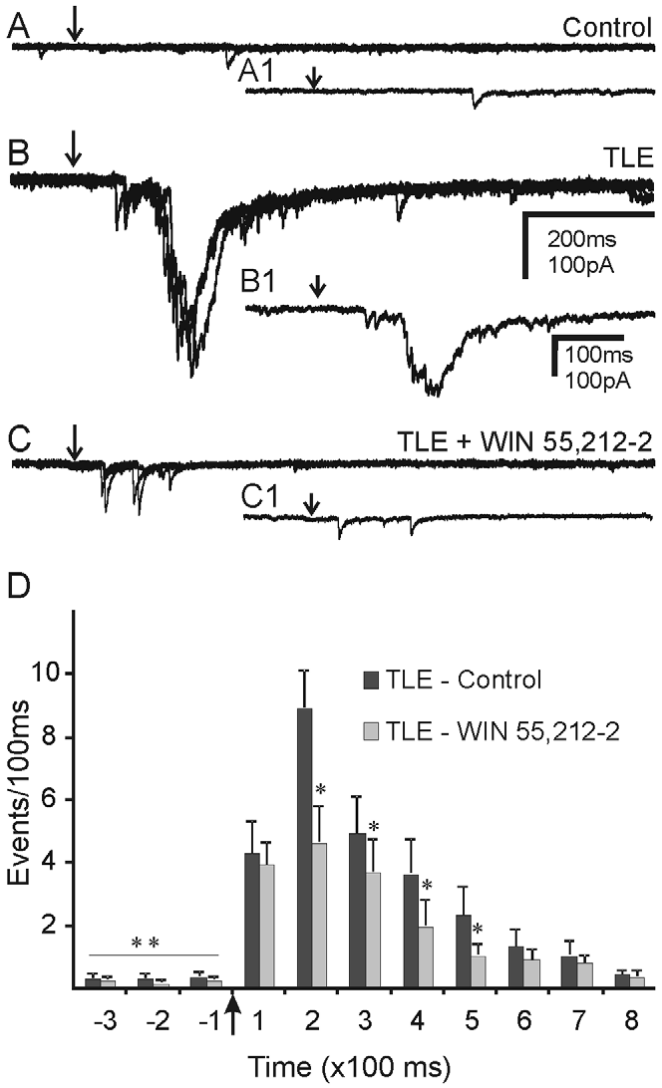
Cannabinoid-mediated attenuation of EPSC bursts evoked by glutamate ‘uncaging’ in the granule cell layer. Sets of 3 overlapping traces showing evoked EPSCs after photolytic release of caged glutamate (250 µM) at the same location in the granule cell layer of control mice and mice with TLE. Arrows indicate the time of uncaging. **A**. Uncaging glutamate in the granule cell layer did not evoke a synaptic response in control mice. **B**. EPSC bursts evoked in pilocarpine-treated mice that survived SE. **C**. In the same cell as **B**, EPSC bursts were attenuated by WIN 55,212-2 (10 µM). **A1**, **B1** and **C1** show a single expanded trace from the group of traces shown in **A**, **B** and **C**. **D**. Grouped data from 6 cells showing the effect of WIN 55,212-2 on EPSC bursts evoked by photolysis of caged glutamate. Glutamate uncaging increased EPSC frequency over baseline; ** indicate significant difference in EPSC frequency before and after photolysis (p<0.05). * indicate a significant reduction in EPSC frequency after application of WIN 55,212-2 during comparable post-photolysis 100 ms time periods (p<0.05).

To more directly test the hypothesis that activity of granule cells contributed to the enhanced sEPSCs in other granule cells in pilocarpine-treated mice that survived SE, neurons in the granule cell layer were selectively stimulated using focal glutamate photolysis [8], [12]. sEPSCs were measured 5 s prior to uncaging and in 100 ms bins for 5 s after photostimulation. Glutamate was photolytically uncaged in at least six positions within the granule cell layer and at additional positions in the hilus and CA3 area. Uncaging glutamate in multiple positions within the dentate gyrus resulted in no change in sEPSC frequency in granule cells from control mice (n = 7; Fig. 7A). However, uncaging glutamate on granule cells in pilocarpine-treated mice that survived SE resulted in a transient increase in EPSC frequency from a background frequency of 3.4±1.1 Hz to a peak frequency of 78.18±8.91 Hz 100–200 ms after uncaging (n = 11; Fig. 7B). Uncaging glutamate in the hilus or CA3 area (n = 2–3 sites per recording) did not evoke a synaptic response in granule cells from either control (n = 7) or pilocarpine-treated mice (n = 11). Similar to observations in rat models of TLE [12], activation of granule cells at a distance from the recorded cell increased EPSCs in granule cells in pilocarpine-treated mice that survived SE, but not in controls.

### Effect of CB1R activation on photolysis-evoked EPSCs

The effect of WIN 55,212-2 was examined on photolysis-evoked EPSC bursts in animals that survived pilocarpine-induced SE. In these cells, WIN 55,212-2 (10 µM) attenuated photolysis-evoked EPSCs in 5 of 6 cells (Fig. 7C,D). The mean number of evoked EPSCs measured 100-200 ms after uncaging was decreased from 89.44±12.28 to 46.67±11.42 Hz by WIN 55,212-2 in these cells. Thus, WIN 55,212-2 attenuated EPSC activity that was driven by action potentials generated in other granule cells (n = 5; p<0.05).

### CB1R Expression

**Figure 8:**
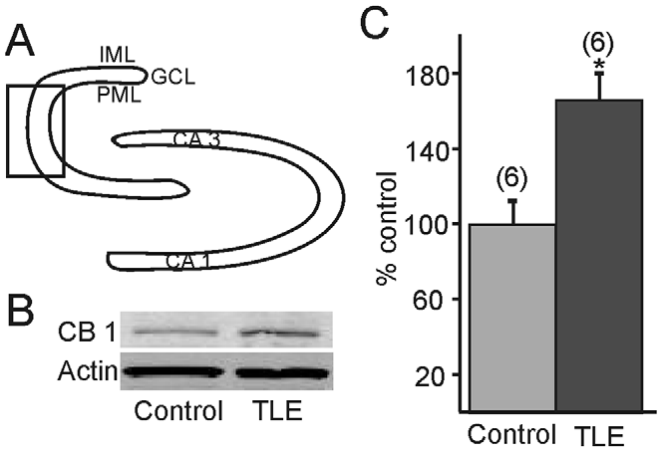
Western blot detection of cannabinoid type 1 receptor (CB1R) expression. **A**. Diagram of dentate gyrus illustrating the area that was micro-dissected (box) for analysis. **B**. Western blot showing CB1R expression in pilocarpine-treated mice that survived SE and developed TLE compared to untreated mice. Actin was used as the loading control, which did not change significantly. **C**. Graph showing cumulative (60%) increase in CB1R expression in mice with TLE versus controls (n = 6; p<0.05). * indicates significance. IML, inner molecular layer; GCL, granule cell layer; PML, polymorphic layer.

Since effects were seen in animals with mossy fiber sprouting but not control animals, we reasoned that the newly sprouted axon terminals expressed CB1R and this may result in an increase in CB1R protein expression in the dentate gyrus of pilocarpine-treated mice that survived SE. The dentate gyrus was microdissected to include the granule cell layer and the molecular layer in controls and pilocarpine-treated mice that survived SE. A discrete band at ∼56 kD, corresponding to the molecular weight of the CB1R receptor was identified by Western blot. Density analysis indicated that the protein binding in tissue from pilocarpine-treated mice that survived SE was increased by 60% over that observed in tissue from control mice (n = 6; p<0.05; Figure 8).

## Discussion

In animal models of TLE and in many patients, a permanent change in neuronal circuitry occurs that renders the brain more susceptible to seizure generation [1], [2], [6]. Among the hallmarks of TLE are hippocampal sclerosis, neuron loss, and synaptic reorganization in the dentate gyrus [36], [37], [38], [39], [40], [41], [42]. We used a pilocarpine treated mouse model for our experiments, which has many of the same characteristic features as seen in human tissues, and is very similar to inducible TLE models in rats [7], [8], [43]. That is, pilocarpine-induced status epilepticus triggers a cascade of events that includes selective neuron loss, gliosis, mossy fiber sprouting with concomitant synaptic reorganization in the dentate gyrus, and, after a period of a few days, development of spontaneous recurrent behavioral seizures ensues [1], [7]. Mice that survived pilocarpine-induced SE had robust mossy fiber sprouting into the inner molecular layer of the dentate gyrus. These animals also developed spontaneous seizures by about 3 weeks after pilocarpine treatment [7]. Whole-cell patch-clamp recordings performed in the absence of Mg^2+^ to allow excitatory network activation [15], [46], [47] and in the presence of bicuculline to suppress GABA_A_ receptor-mediated inhibition revealed epileptiform activity, which included barrages of EPSCs in pilocarpine-treated mice that survived SE. Under identical recording conditions, such activity was not seen in normal animals or in mice that did not undergo SE after pilocarpine injection, suggestive of a reorganized synaptic circuit in the dentate gyrus of epileptic mice.

Although the relationship between synaptic reorganization and spontaneous seizure development is indirect, analysis of the cellular and synaptic changes observed in this and other models may be relevant to understanding the processes that contribute to epileptogenesis. Synaptic reorganization is hypothesized to contribute to seizure susceptibility, but the new connections also may represent a potential target for attenuating activity, which is unique to the reorganized circuit. Most antiepileptic drugs were developed in unaltered brain systems, and thus they do not target the abnormal synaptic circuitry that develops in TLE. Identifying pharmacological targets on the newly-formed axon terminals may result in development of new strategies for suppressing seizures [44], [45]. Since CB1R are generally considered to be located on synaptic terminals in the dentate gyrus, we assessed effects of activating those receptors on glutamatergic terminals in a murine model of TLE. Effects on GABA release have yet to be assessed in this model, but the present results suggest that CB1R are expressed functionally in excitatory circuits to a much greater degree in the reorganized dentate gyrus than in control mice. The robust CB1R-mediated reduction of mEPSC frequency in slices from epileptic mice suggests a presynaptic location for the receptors on glutamatergic synaptic terminals. Input resistance and resting membrane potential were not altered by CB1R activation, further suggesting that postsynaptic changes in cellular activity did not account for the suppression of glutamate release. Binding CB1R suppressed secondary population afterdischarges subsequent to antidromic activation of the granule cells. Secondary discharges in this and other models of TLE have been shown to be driven by action potentials in neurons contained within the slice that are activated by the antidromically-induced action potential in granule cells [8], [9]. Such afterdischarges are not typically seen in controls, especially when the entorhinal cortex has been completely excised from the slice, as in the present studies. This suggests that the circuit upon which the agonists acted was part of the new recurrent excitatory circuitry that forms after hippocampal sclerosis and development of TLE. These results are consistent with the hypothesis that CB1R activation suppresses recurrent excitatory activity in epileptic mice, which does not occur in the granule cell layer of control mice.

In addition to suppressing circuit activation induced by antidromic stimulation of mossy fibers, cannabinoid agonists reduced EPSCs evoked by glutamate photolysis stimulation of other granule cells. These data are consistent with the hypothesis that the cannabinoid agonists mediated suppression of glutamate release subsequent to binding CB1R located on sprouted glutamatergic terminals of the reorganized recurrent excitatory circuit. This binding was sufficient to reduce network excitability. In normal animals, CB1R binding can inhibit glutamatergic input arising from mossy cells [48]. Mossy cells and CA3 pyramidal cells normally project onto granule cells [49], [50], and CB1R-mediated inhibition of these terminals might also contribute to the enhanced effect in animals with TLE, especially if terminals of surviving cells sprout. However, glutamate photolysis in either the CA3 pyramidal cell layer or the hilus failed to elicit synaptic responses in the dentate gyrus, indicating that such connections were not prominent in our slices. It remains possible that sprouted axons of glutamatergic inputs from other areas contribute to the increased excitability, and that CB1R expression on such terminals may also play a role in the effect of cannabinoids. However, sprouted mossy fibers appear to play the major role in this new circuitry [8], [10], [13] and CB1R-mediated responses were obvious when granule cells were activated.

The present data suggest a presynaptic location of CB1R, consistent with other studies indicating that CB1R are present on pre-synaptic nerve terminals elsewhere in the hippocampal formation [24], although they are typically thought to be located on terminals of GABAergic neurons [51], [52],. Therefore, the usual action of cannabinoids would be to disinhibit granule cells, an action which might be expected to increase the likelihood of seizures. However, CB1R agonists suppress seizure activity in rats that have developed TLE[31], [53], consistent with their location in glutamatergic circuits. Although the effect observed here of CB1R agonists on connections in the dentate gyrus represent only indirect evidence of CB1R-mediated anticonvulsant activity, the response we observed in slices is consistent with the effects seen in vivo.

In the normal dentate gyrus, CB1R on terminals of hilar mossy cells likely accounts for their presence in the inner molecular layer and these receptors are believed to mediate mossy cell terminal-specific DSE in granule cells [48]. We found no significant effects of CB1R activation on overall sEPSCs or mEPSCs in control mice, but effects of CB1R activation specifically on mossy cell inputs were not tested. Effects of cannabinoid agonists on putative granule cell-to-granule cell connections were inhibited, but only in animals with mossy fiber sprouting. Endogenous cannabinoid release (e.g., anandamide, 2-AG) is thought to be activity-dependent, and levels of endogenous cannabinoids are increased during seizures [31], [54]. We did not directly assess activity of endogenous cannabinoid release in the present study, but application of AM251 alone had little effect on EPSCs. Endogenous cannabinoid release may not necessarily be chronically elevated in slices from epileptic mice, even though glutamatergic synaptic activity was enhanced. Also, our recording conditions may not have been optimal for detecting endogenous activity. It will be of interest to determine if evoked endogenous cannabinoid release is altered in the dentate gyrus of mice with TLE.

The density of CB1R has been reported to be down regulated in the dentate gyrus in TLE patients and some animal models [29], [30]. The loss of hilar mossy cells and GABAergic interneurons during the development of TLE in similar models [55] and the concomitant loss of CB1R associated with these nerve terminals could contribute to a reduction in the density of CB1R observed in immunohistochemical analyses. Increases in CB1R expression were shown in the hippocampus, if not in the dentate gyrus, in other models of TLE [31], [56], [57], and receptor levels appear to change biphasically in the whole hippocampus over time after SE, being reduced initially and then increased over several weeks [29]. Notably, different CB1R antibodies show different distribution patterns, depending on the binding epitope [58], [59]. We used Western blot to minimize this issue and detected an increase in the total CB1R protein expression in the temporal half of the isolated dentate gyrus of pilocarpine-treated epileptic mice with robust mossy fiber sprouting. Receptors on axon terminals arising from a variety of areas could contribute to the increase in overall expression we observed, including on GABAergic terminals, so electron microscopic analyses may be necessary to demonstrate conclusively an increase in receptors associated with mossy fibers. However, while spatial resolution is limited with the Western blot technique, these results were consistent with our electrophysiological data indicating an increase in CB1R-dependent suppression of local glutamatergic synaptic connections, probably between granule cells. This suggests that CB1R could be made in the cell body of the granule cells and be trafficked to the terminals. Message for CB1R in granule cells is normally low in the dentate gyrus, but not absent [26], [60], [61], and seizures or SE can cause up-regulation of a variety of genes [62]. Whether CB1R message is up regulated after SE in this model is not known but our data would be consistent with that hypothesis.

Many cannabinoid agonists bind both CB1R and CB2R, and AEA is also an agonist at TRPV1 receptors, activation of which can enhance release of neurotransmitter by increasing intracellular concentrations of Ca^2+^ [63]. TRPV1 receptors have been shown to exist in the dentate gyrus [63]. The inhibitory effect of AEA was mimicked by WIN 55,212-2 and 2-AG, which are not known to efficiently bind TRPV1 receptors. TRPV1 activation generally increases glutamate release; it seems unlikely that TRPV1 contributes to the decrease in synaptic activity. Notably, an AEA-induced transient increase in EPSC frequency was observed in a subset of neurons and involvement of TRPV1 in that response has been identified [64]. Finally, inhibitory effects of CB1R agonists were blocked by AM251, a relatively selective CB1R antagonist/inverse agonist. Reduction of the frequency of glutamate-evoked EPSCs and antidromically-evoked secondary population activity by CB1R activation suggests that the remodeled circuit expresses functional CB1R. In addition to blocking CB1R-mediated effects, unmasking of TRPV1-mediated effects of AEA in the presence of CB1R antagonists might be expected to transiently enhance recurrent excitation in the dentate gyrus in some cases. This may be relevant to a recent report indicating recurrence of seizures in a TLE patient treated with a CB1R antagonist [33].

Both endogenous and synthetic cannabinoid agonists reduced the frequency of the excitatory glutamatergic events in addition to blocking the epileptiform barrages originating from activity in other granule cells in the synaptically reorganized dentate gyrus of mice with TLE. The ability of cannabinoids to block epileptiform discharges in this model of TLE could have significant therapeutic implications, particularly in patients with mesial temporal sclerosis and mossy fiber sprouting. However, further study is needed to understand the interactions between cannabinoids and other receptor targets as well as cannabinoid effects in other brain areas affected in TLE or in other TLE models, where axon sprouting may be significant but not as robust as in the pilocarpine-treated mouse.
